# Supplementary figures and images for: LENG8 mediates RNA nuclear retention and degradation in eukaryotes
Source: bioRxiv. 2025 Aug 16:2025.08.14.670437. Preprint. [Version 1] doi: 10.1101/2025.08.14.670437 (PMC12363940; doi:10.1101/2025.08.14.670437)

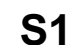

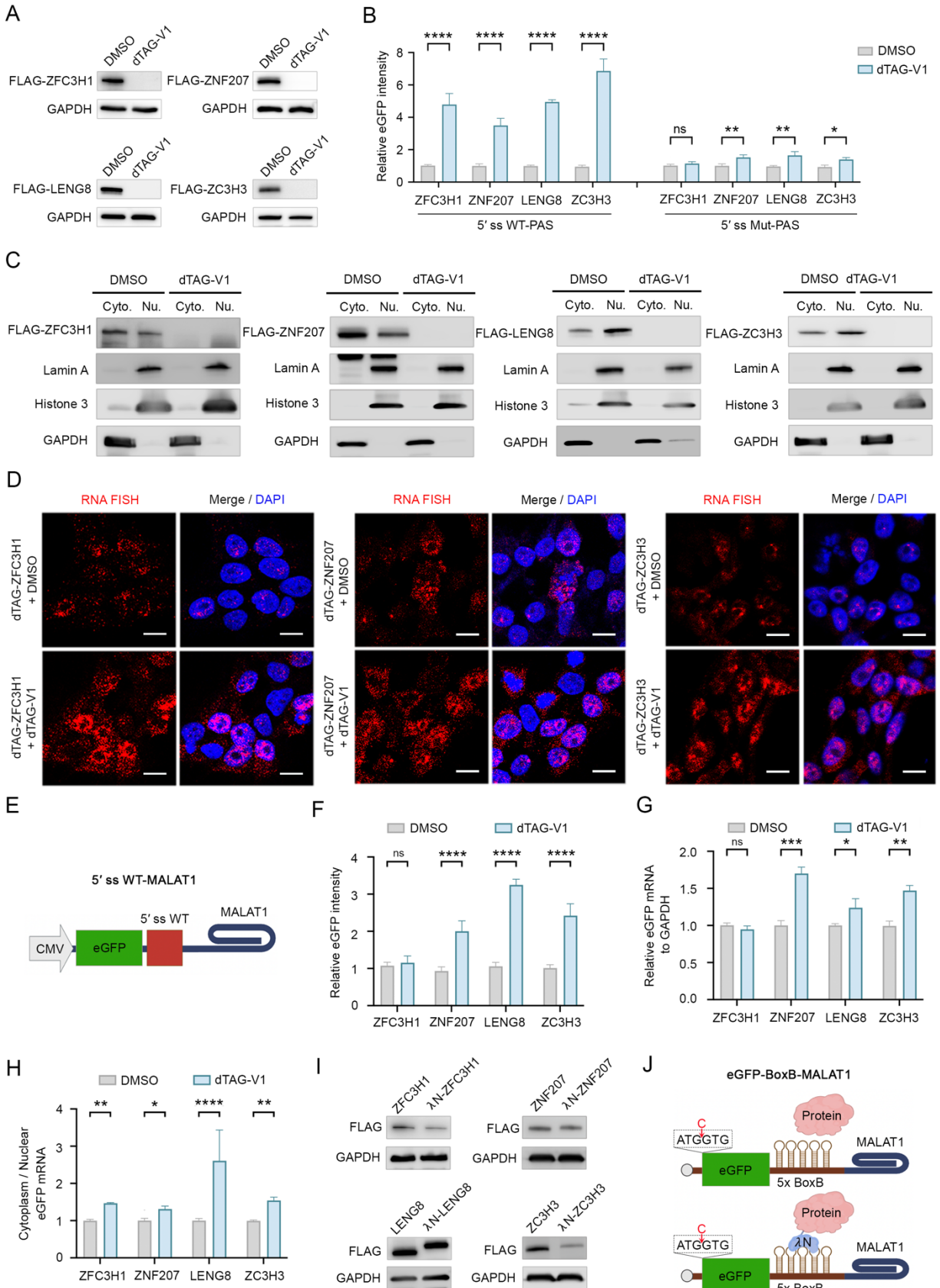

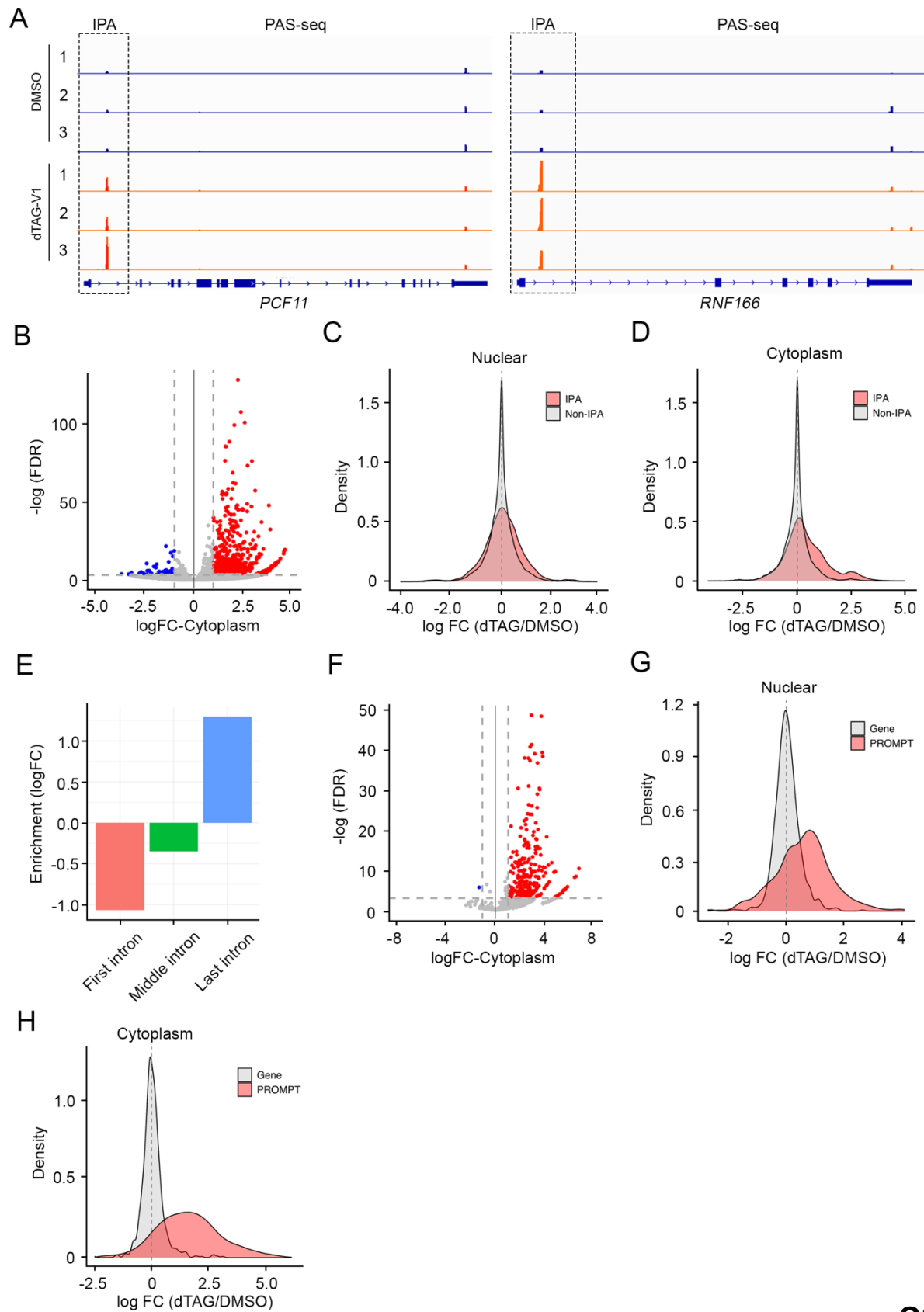

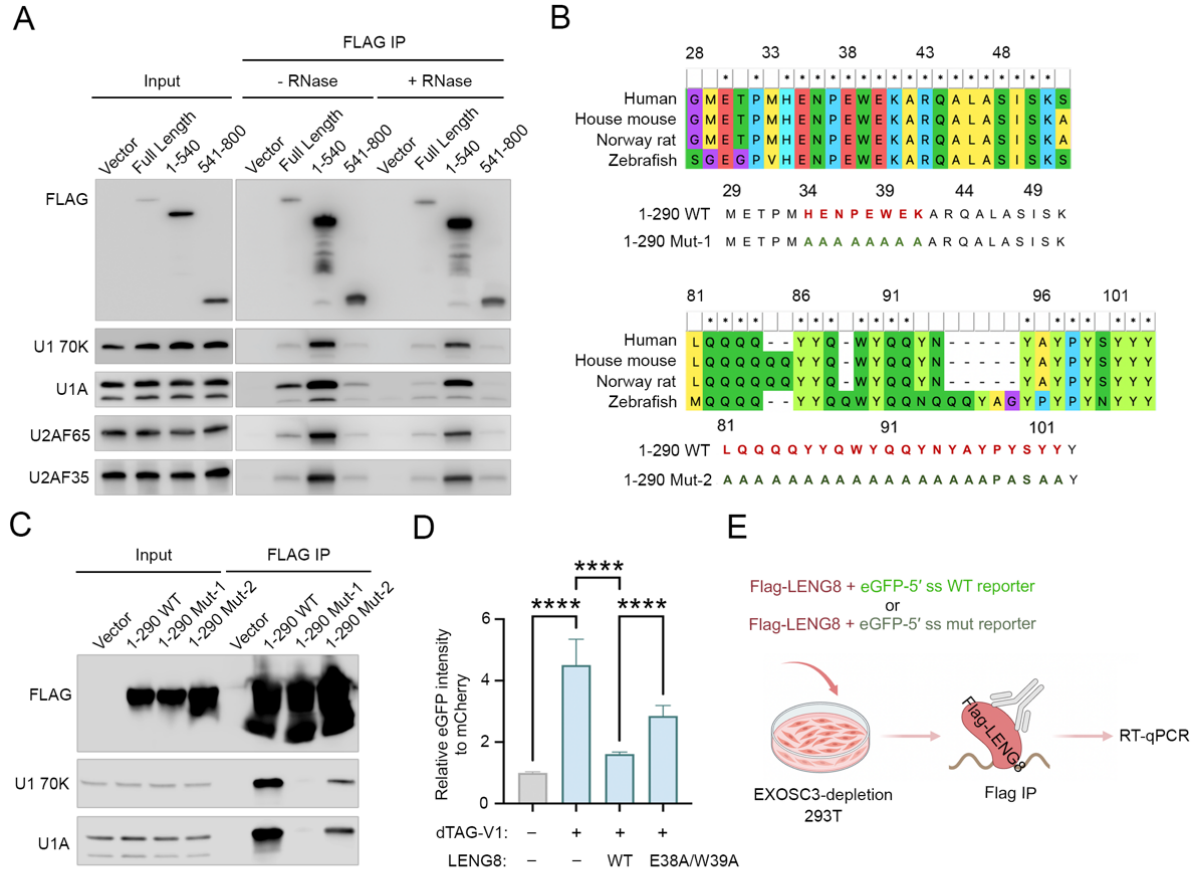

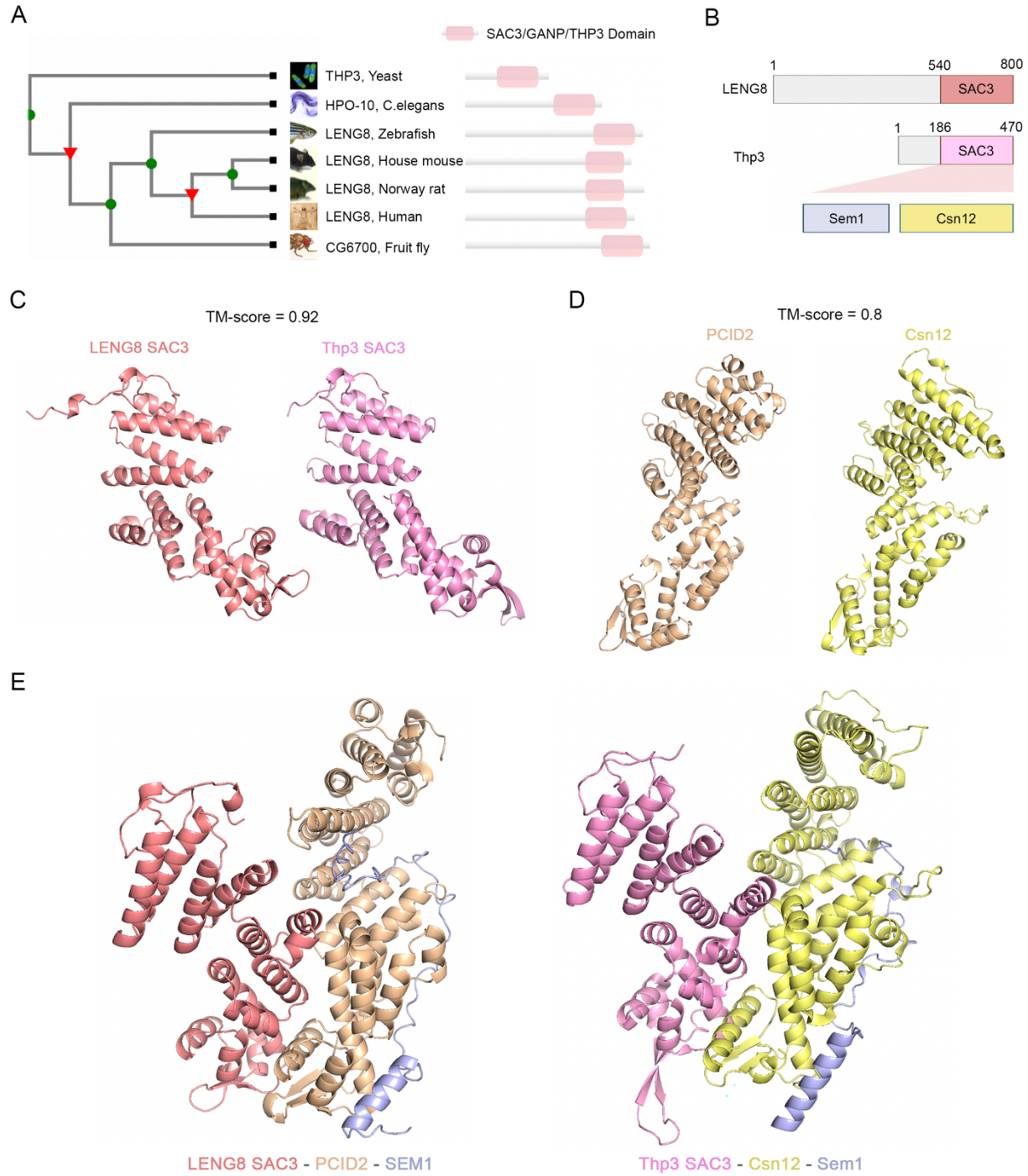

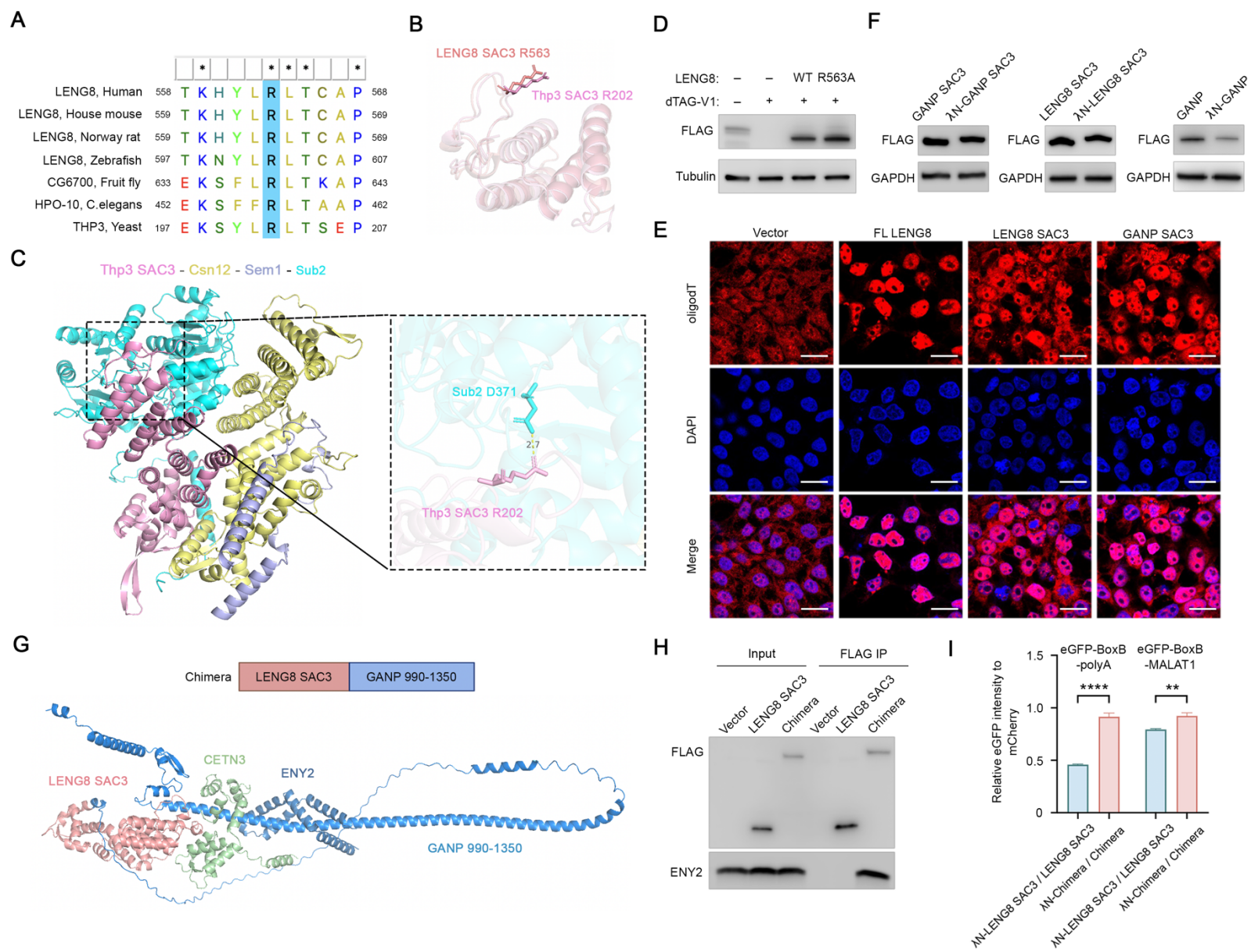

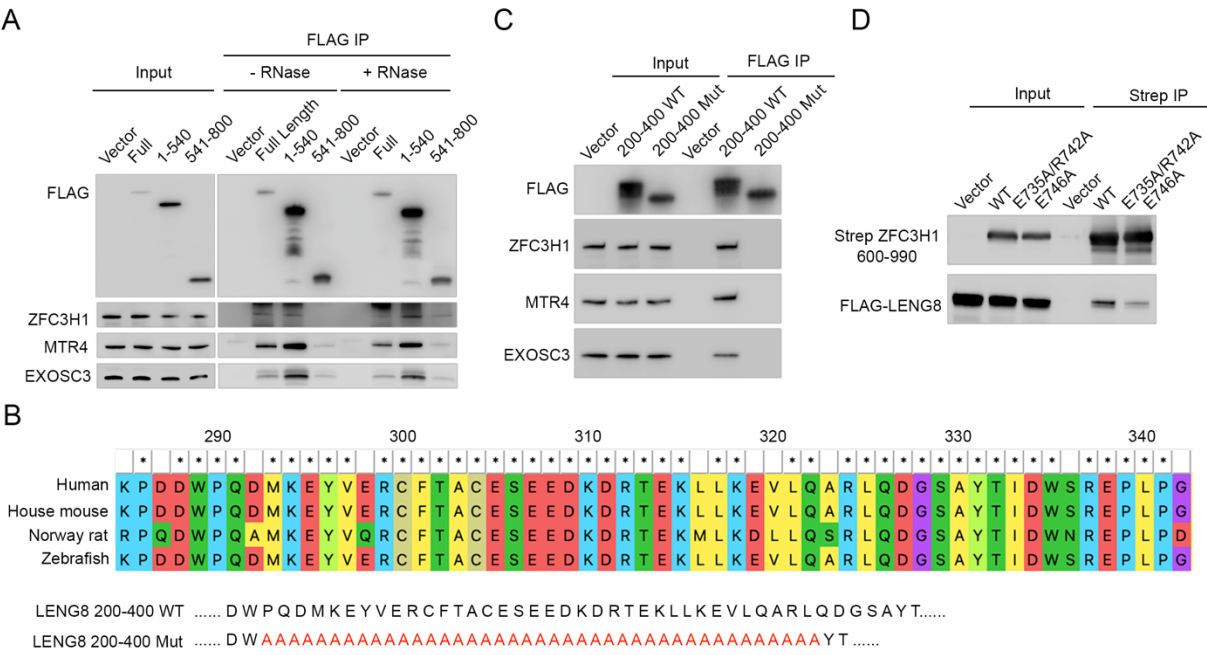

Supplement: 1 [file NIHPP2025.08.14.670437V1-supplement-1.pdf]
